# Neck of femur fractures in the over 90s: a select group of patients who require prompt surgical intervention for optimal results

**DOI:** 10.1007/s10195-013-0248-9

**Published:** 2013-07-17

**Authors:** K. S. Hapuarachchi, R. S. Ahluwalia, M. G. Bowditch

**Affiliations:** 1 New Zealand Orthopaedic Training Program, Wellington, New Zealand; 2 Department of Orthopaedics, Chelsea and Westminster Hospital, London, UK; 3 Ipswich Hospitals NHS Trust, Ipswich, Suffolk

**Keywords:** Fracture neck of femur, Nonagenarians, Timing of surgery

## Abstract

**Background:**

Patients in the extremes of old age with a femoral neck fracture represent a challenging subgroup, and are thought to be associated with poorer outcomes due to increased numbers of comorbidities. Whilst many studies are aimed at determining the optimum time for surgical fixation, there is no agreed consensus for those over 90. The aim of this study is to report the surgical outcome of this population, to understand the role surgical timing may have on operative outcomes using the orthopaedic POSSUM scoring system and to identify whether medical optimization occurs during the period of admission before surgery.

**Materials and methods:**

We conducted a prospective observational study; data was collected from two district general hospitals over 32 consecutive months. All patients aged 90 and above who were deemed suitable for surgical fixation were included. Each one had their orthopaedic POSSUM score calculated at admission and at surgery, using their computerised and paper medical records. Assessment of outcome was based on morbidity and mortality at 30 days.

**Results:**

A total of 146 consecutive patients above the age of 90 underwent surgery and were followed. The average age of the patients was 93 years, 123 (84 %) were female and 23 (16 %) male. Sixty-one patients were operated on within 24 h from admission, 52 patients within 24 and 48 h and 33 had surgery after 48 h from admission. In total, 21 deaths (14.4 %) were recorded and 81 patients (55.5 %) had a post-operative complication within 30 days. The orthopaedic POSSUM scoring system predicted 30-day mortality in 23 patients and morbidity in 83 patients. This gave observed to predicted ratios of 0.91 and 0.98 respectively. Overall, there was a small improvement in physiological scores taken just prior to surgery compared to those at admission. Mortality and morbidity rates were higher for those operated on or after 24 and 48-h cutoffs compared to those proceeding to surgery within 24 h (*P* = 0.071 and *P* = 0.021 respectively and *P* = 0.048 and *P* = 0.00011 respectively). When stratified according to their POSSUM scores, patients with scores of 41+ and surgery after 48 h had a significantly higher mortality rate than if they had surgery earlier (*P* = 0.038). Morbidity rates rose after 24 h of surgical delay (*P* = 0.026). Patients with a total POSSUM score between 33 and 40 exhibited a higher morbidity after a 24-h delay to surgery (*P* = 0.0064).

**Conclusion:**

As life expectancy increases, older patients are becoming commoner in our hospital systems. We believe the orthopaedic POSSUM scoring system can be used as an adjuvant tool in prioritising surgical need, and allow for a more impartial evaluation when changes to practice are made. Our findings show that timing of surgery has an important bearing on mortality and morbidity after hip surgery, and older patients with higher orthopaedic POSSUM scores are sensitive to delays in surgery.

## Introduction

Fractures of the femoral neck in the elderly are common and are a devastating injury that extends far beyond the musculoskeletal trauma, with significant long-term consequences for the quality of life of both patients and carers. Patient care is complex, requiring multidisciplinary teams and integrated care pathways. Patients above the age of 90 represent a challenging subgroup as they have a number of concurrent medical comorbidities, and are susceptible to postoperative complications and poorer outcomes [1].

The timing of surgery in the elderly following a fracture of the femoral neck has long been debated. Whether surgical delay contributes to a poorer outcome remains controversial [2]. Evaluating the literature in this area is difficult because of differing methodologies, complex case mixes and the varying structures of trauma care, e.g. orthogeriatrician care [3].

Thus, a scoring system is needed with the ability to predict poor outcomes following surgery and to provide an objective measure of effective treatment where a multitude of different variables exist. Previous work has identified postoperative complications after neck of femur fractures to have a resultant effect on the long-term morbidity and mortality of the patient [2]. The physiological and operative severity score for the enumeration of mortality and morbidity (POSSUM) is a scoring system that predicts postoperative morbidity and mortality, taking into account the patient’s physiological as well as surgical factors [4]. Originally designed to assess outcome after general surgery, the POSSUM scoring system has been modified for orthopaedic surgery and validated by Mohamed et al. [5]. We hypothesize that the orthopaedic POSSUM scoring system could predict later mortality and morbidity in hip fractures and help in prioritizing early fracture fixation in this group of patients.

The aim of this study is to report the outcome of patients above the age of 90 sustaining a fracture of the femoral neck, and assess the use of the orthopaedic POSSUM to understand the role of timing delays on outcomes following fracture fixation.

## Materials and methods

### Patient population

This was a prospective observational study; data was collected from February 2005 to September 2007. All patients aged 90 and above who underwent surgical fixation for a fractured neck of femur at two district general hospitals in the UK were included. Patients were cross-referenced with the individual trust’s hip database, admissions list and local audit databases to ensure complete capture. Local ethical approval was sought and granted as audit; the study was performed in accordance with the ethical standards of the 1964 Declaration of Helsinki as revised in 2000. The need for informed consent was waived since the rights and interests of the patients would not be violated and their privacy and anonymity assured by the study design.

One hundred and forty-six consecutive patients above the age of 90 had surgical fixation of a fractured neck of femur over a period of 34 months. None of these patients were excluded from the study and all patients were followed up to 1 year post operatively. The average age of the patients was 93 years, 123 (84 %) were female and 23 (16 %) male. All patients were treated according to local hospital protocol, thus aiming to undertake surgery within 24 h from admission. Surgery was performed in a laminar flow theatre and all patients receive prophylactic antibiotics and thromboprophylaxis with low molecular weight heparin. A consultant orthopaedic surgeon would recommend the type of surgery, and an anaesthetist in conjunction with the orthogeriatricians assessed all patients for fitness for surgery.

Each patient had their orthopaedic POSSUM score calculated as described by Mohamed et al. (2002) [5]. Both physiological scores and an operative severity score were collated. The physiological score is divided into 12 categories and the operative severity score into 6 categories. Each category is graded on an exponentially increasing value (Table 1). The physiological POSSUM score was calculated at admission and immediately prior to surgery and compared to analyse any change in order to achieve medical optimization. The operative severity score was calculated from the operative notes and histology results if available; fractures were assumed not to be pathological if no histology was found.

**Table 1:** Physiological and operative severity assessment in the orthopaedic POSSUM system

	Physiologic ill score					Operative severity score			
	1	2	4	8		1	2	4	8
Age (years)	<60	61–70	>71		Magnitude	Minor	Inter	Major	Major+
Cardiac signs	Normal	On cardiac drugs or steroid	Oedema Warfarin	Raised JVP^a^	Number of operative variables within 30 days	1		2	>2
Chest radiograph	Normal		Borderline cardiomegaly	Cardiomegaly	Blood loss per operation (ml)	<100	101–500	501–999	>1,000
Resp signs	Normal	SOB^b^ exertion	SOB stairs	SOB rest	Contamination	None	Incised would, i.e. stab	Minor contamination or necrotic tissue	Gross contamination or necrotic tissue
Chest radiograph	Normal	Mild COAD^c^	Mod COAD	Any other change	Presence of malignancy	None	l_0_	Node metastases	Distant metastases
Systolic BP (mmHg)	110–130	131–170 100–109	>171 90–99	<89	Timing of operation	Elective		Emergency Resuscitation possible <48 h	Emergency Immediate <6 h
Pulse (/min)	50–80	81–100 40–49	101–120	>121 <39					
Como score	15	12–14	9–11	<8					
Blood urea (mmol/1)	<7.5	7.6–10	10.1–15	>15.1					
Blood Na (mmol/1)	>136	131–135	126–130	<125					
Blood K (mmol/l)	3.5–5	3.2–3.4 5.1–5.3	2.9–3.1 5.4–5.9	<2.8 >6					
Hb (g/100 ml)	13–16	11–12.9 16.1 to 17	10–11.4 17.1–18	<9.9 >18.1					
White cell count (×1012/1)	4–10	10.1–20 3.1–3.9	>20.l <3						
ECG	Normal		AF^d^ (60–90)	Any other change					

^a^Jugular venous pressure

^b^Shortness of breath

^c^Chronic obstructive airways disease

^d^Artrial fibrillation

Predicted mortality and morbidity was calculated from the physiological score taken immediately prior to surgery and not at the time of admission, since this represented actual patient health at time of surgery. Assessment of actual outcome was based on morbidity and mortality at 30 days with the exact definitions of postoperative complications as described by Copeland et al. [4]. The assessment of the POSSUM score was made by calculating an observed to predicted ratio where 1 represented parite between the tests.

To assess any delay in surgery we defined time to surgery as the time from admission to the time of the operation. It was divided into three categories; surgery within 24 h (early), between 24 and 48 h (intermediate) and after 48 h (late) from admission. To compare patients proceeding to surgery at different time points we used the *χ*^2^ test to compare mortality and morbidity against timing of surgery at both the 24 and 48-h cutoffs and also when the patients were stratified according to their total orthopaedic POSSUM scores. If the expected frequency was less than 5 when calculating the *χ*^2^ statistic, then the Fisher exact probability test was used instead, being more accurate for smaller sample sizes.

For a more impartial evaluation of surgical timing and its effect on mortality and morbidity, a comparison between patients of equal predicted morbidity and mortality rates at admission and different surgical time points was conducted to exclude confounding factors such as differing physiological compromise and medical comorbidities. We stratified the patients by their orthopaedic POSSUM score at admission and then compared the predicted mortality and morbidity depending on surgical time.

## Results

In total, we observed 21 deaths (14.4 %) within 30 days of surgery and 81 patients (55.5 %) with a postoperative complication. Sixty-one patients were operated on within 24 h (early group) from admission, 52 patients within 24–48 h (intermediate group) and 33 had surgery after 48 h from admission (late group). There were 5 deaths out of 61 patients (8.2 %) in the early group compared to 7 out of 52 patients (13.46 %) in the intermediate group and 9 out of 33 patients (27.3 %) within the late group. Similarly, there were 28 patients (45.9 %) with postoperative complications in the early group compared to 25 patients (48.1 %) in the intermediate group and 28 patients (84.9 %) in the late group.

Stratifying this data into 24-h and 48-h cutoffs, the mortality rose if surgery was delayed for 48 h. (*P* = 0.021, Table 2). The observed morbidity rate was higher for those patients proceeding to surgery between 24 and 48 h compared to those having surgery earlier (*P* = 0.048 and *P* = 0.00011 respectively, Table 2).

**Table 2:** The effect of surgical delay in fracture neck of femur surgery

Observed mortality and morbidity rates at 24 and 48-h cutoffs						
	Delay of surgery			Delay of surgery		
	≤24 h	>24 h	*P* value	≤48 h	>48 h	*P* value
Mortality	8.20 %	18.82 %	0.071	10.62 %	27.3 %	**0.021**
Morbidity	45.90 %	62.35 %	**0.048**	46.90 %	84.8 %	**0.00011**

Bold values indicate statistical significance (*P* < 0.05)

**Fig. 1:**
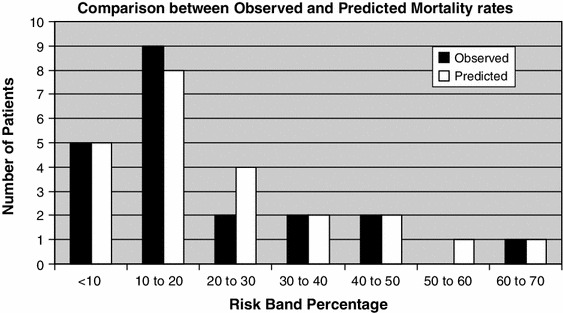
Observed versus predicted mortality stratified according risk bands

**Fig. 2:**
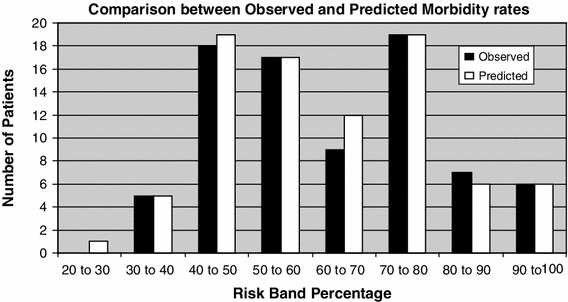
Observed versus predicted morbidity stratified according risk bands: a comparison of actual morbidity and mortality for the individual surgical periods

The POSSUM predicted an overall mortality in 23 patients and morbidity in 83 patients. This gave observed to predicted ratios of 0.91 and 0.98 respectively, making it more accurate at predicting morbidity. Comparison between the POSSUM predictions and observed mortality and morbidity for increasing levels of risk is given in Figs. 1 and 2, showing a close association between the predicted and observed values across all levels of POSSUM risk score that were recorded in this population.

When we looked at the physiological scores taken just prior to surgery and compared them to scores taken at admission, there was a marginal improvement in the 24–48-h group (intermediate) and 48-h (late) group (Table 3). The early group showed a slight deterioration in the POSSUM score (Table 3).

**Table 3:** Comparison of physiological scores taken at admission and at surgery

Orthopaedic POSSUM scores and predicted mortality and morbidity for each group						
	Early (24 h)		Intermediate (48 h)		Late (>48 h)	
	Admission	Surgery	Admission	Surgery	Admission	Surgery
Physiological score	22.934	23.082	23.692	23.558	25.394	24.818

In general there is an increase in the orthopaedic POSSUM score as the patient waits for his or her operation

As there was marginal improvement we tried to assess the cause of delay, even though this was not an original aim of our study. Eighty-five patients were delayed, but only 36 had an identifiable cause and a defined reason for delay. Sixteen were deemed medically unfit, in the majority of cases this was cardiac related, four had a preceding myocardial infarction and three were related to concurrent chest sepsis. Twelve patients were coming off warfarin or another anticoagulant, four patients were waiting further imaging e.g. a chest CT and four were cancelled due to lack of surgical time.

Assessing a delay in surgery by stratifying patients through their admission POSSUM score showed increased predicted mortality if the delay was greater than 48 h. The greatest mortality was seen if the POSSUM score was over 42 (*P* = 0.0092, Table 4). Thus, surgical delay had no effect on mortality as long as the admission orthopaedic POSSUM score was less than 42. If the admission orthopaedic POSSUM score was 40 or above, there was increased risk of morbidity if surgery was conducted 24 h or later post admission (24–48 h delay *P* = 0.026 and 48 h *P* = 0.029, Table 5). However, morbidity only rose when the delay in surgery was greater than 48 h if patients presented with an admission orthopaedic POSSUM score as low as 33 at 48 h (*P* = 0.0064, Table 5). The morbidity or mortality outomes of patients who scored 22–33 were not affected by delays in surgery.

**Table 4:** Orthopaedic POSSUM scores and the stratification of the effect of delay in surgery on patient mortality

Observed mortality rates risk stratified to total orthopaedic POSSUM score						
POSSUM score	Delay of surgery			Delay of surgery		
	≤24 h	>24 h	*P* value	≤48 h	>48 h	*P* value
≤36	8.11 %	8.51 %	0.63	7.69 %	10.53 %	0.50
37–40	12.50 %	35.29 %	0.25	23.81 %	50.0 %	0.31
≥42	6.25 %	28.57 %	0.096	7.41 %	50.0 %	**0.0092**

The evidence indicates that an orthopaedic POSSUM score of >42 is indicative of increasing mortality at 48 h

Bold value indicates statistical significance (*P* < 0.05)

**Table 5:** Morbidity I found to be predicted by lower POSSUM scores and positively associated with any delay in surgery

Observed morbidity rates risk stratified to total orthopaedic POSSUM score						
POSSUM score	Delay of surgery			Delay of surgery		
	≤24 h	>24 h	*P* value	≤48 h	>48 h	*P* value
≤32	29.41 %	21.43 %	0.47	20.00 %	50.00 %	0.16
33–39	46.15 %	60.42 %	0.24	47.46 %	86.67 %	**0.0064**
≥40	61.11 %	91.30 %	**0.026**	68.97 %	100.00 %	**0.027**

Bold values indicate statistical significance (*P* < 0.05)

## Discussion

Our first objective was to define the outcome of nonagenarians who sustain a neck of femur fracture and go on to have surgery. As a group, we found morbidity and mortality was higher in this than in other age groups. It has been shown that age as well as male sex and renal problems are factors that increase mortality at 30 days [6]. The overall mortality for those over 60 with an acute hip fracture ranges from 9.6 % at 30 days to 33 % at 1 year, or up to 19 % at 19 days [7, 8]; our figures sit between these. As our results are not dissimilar to those for anyone over 60 this suggests there may well be self-selection for a hardened patient within the group. Thus, age should not be a barrier, but should be considered in the type of treatment offered.

Our POSSUM results show a 0.95 correlation with observed mortality. We observed a rise in POSSUM score with surgical delay; thus patients operated after the 24 and 48-h cutoffs had higher observed and predicted mortality and morbidity rates than those who underwent early surgery; with those who had poorer POSSUM scores doing worse. These results may be attributable to increased catabolism, aggravated by the prolonged fasting, delay and pain. Treatment with analgesics has little inhibitory effect on this reaction [8]. The ensuing insulin resistance will accelerate the process of muscle loss and may propagate weakness [9, 10], leading to an increase in time to discharge and recovery of mobility. As patients are on bed rest there is a theoretically increased risk of bed rest-related complications such as thromboembolism, urinary tract infections, atelectasis, and pressure ulcers [11] Similarly, the onset of delirium is common with hip fracture in the elderly and known to be directly related to a waiting time of more than 48 h for surgery [12].

We found a POSSUM score of 32 or less would tolerate a delay of up to 48 h better, without increased morbidity or mortality. If the POSSUM score rose up to 39, then surgery after 48 h seems to increase morbidity. We believe a POSSUM score of 42 or greater should always have surgery as soon as possible: within 24 h if possible, as these patients seem to have the highest predicted mortality and are more sensitive to the detrimental effects of delayed surgery.

It is thus imperative to either improve the POSSUM score in the period before surgery to prevent increased mortality, and reduce it further to less than 33 to prevent significant increases in morbidity. If this cannot be done for whatever reason then it would seem sensible to conduct surgery within the 24-h period to ensure the best outcome if deemed safe. Simunovic et al. [13] have shown in their unadjusted estimates that early surgery significantly reduces the risk of 1-year mortality by 45 %.

Since individuals with lower admission POSSUM scores tolerate surgical delay better, this may help in prioritizing people and emphasise optimizing people as soon as possible. Targeting patients in this way could yield health cost benefits, and correlates with prior studies suggesting that age is an important contributing factor affecting mortality in patients whose surgery is delayed. Thus, sicker patients on admission could undergo surgery immediately [14, 15].

Overall, we saw no improvement in POSSUM score from admission to time of surgery. This may reflect the model of medical care at the time of this study (a liason vs a proactive system). A recent randomised controlled trial (RCT) involving early and continued orthogeriatrician input showed a significant reduction in inpatient mortality and a trend to reduction in 12-month mortality [16] as well as a reduction in length of stay following the intervention. This may have prevented some of the delays in surgery in this study e.g. warfarin cessation and INR reduction [17, 18]. We would concur with British Orthopaedic Association guidelines suggesting these fractures are best managed by a full time consultant or staff grade physician on a fracture ward, providing daily medical care and advice in the perioperative management of older patients with hip fractures [19]. Also, perioperative management undertaken by experienced anaesthetic personnel [20, 21] will avoid unnecessary investigations such as echocardiography, which take time. Guidelines from the American College of Cardiology and the American Heart Association 2007 for investigation of cardiac disease for non-cardiac surgery do not support the use of additional investigations in most patients [22, 23].

One study expressed concerns that POSSUM itself over-predicts mortality and morbidity [24]; their data only reflected a single centre. Our experience indicates that their results could be at variance with national/international experience and the POSSUM could be acceptable at predicting outcomes in nonagenarians and prioritizing patients to ensure timely surgery and preventing further deterioration while waiting for surgery. However, we advocate further studies to test the level at which a POSSUM score becomes significant to validate it as part of the systematic tool used to prioritize emergency trauma lists. The current study is limited by a small number of patients in the over 90’s group. We have studied one model of care and not assessed the implications of surgical factors (such as the type of operation and length of surgery and surgical grade, out of hours surgery and implant used) as well as specific patient factors (including specific comorbidities, and mobility status). Repeating the study with different medical models could assess the sensitivity of the POSSUM.

In conclusion, this study looked at one model of care, with complete data on a large consecutive series of patients with 100 % follow-up for mortality statistics reflecting everyday clinical practice in the UK. We have shown that the orthopaedic POSSUM can accurately predict mortality and morbidity and may be used as a tool in prioritizing patients for early surgery and help in further studies.
